# Adverse Outcomes Associated With Interleukin-6 in Patients Recently Hospitalized for Heart Failure With Preserved Ejection Fraction

**DOI:** 10.1161/CIRCHEARTFAILURE.122.010051

**Published:** 2023-03-10

**Authors:** Leanne Mooney, Colette E. Jackson, Carly Adamson, Alex McConnachie, Paul Welsh, Rachel C. Myles, John J.V. McMurray, Pardeep S. Jhund, Mark C. Petrie, Ninian N. Lang

**Affiliations:** School of Cardiovascular and Metabolic Health (L.M., C.E.J., C.A., P.W., R.C.M., J.J.V.M., P.S.J., M.C.P., N.N.L.), University of Glasgow, United Kingdom.; Robertson Centre for Biostatistics (A.M.), University of Glasgow, United Kingdom.

**Keywords:** heart failure, inflammation, interleukin, natriuretic peptide, brain, tumor necrosis factor

## Abstract

Inflammation may play a role in the pathophysiology of heart failure with preserved ejection fraction. We examined whether circulating levels of interleukin-6 identify patients at greater risk of adverse outcomes following hospitalization with heart failure with preserved ejection fraction.

**Methods:**

We assessed relationships between interleukin-6 (IL-6) tertiles (T1-3) and all-cause death, cardiovascular death, and subsequent heart failure hospitalization (sHFH) in 286 patients recently hospitalized with heart failure with preserved ejection fraction. Associations between IL (interleukin)-6 and outcomes were examined in a Cox-regression model adjusted for risk factors including BNP (B-type natriuretic peptide). Biomarkers including hsCRP (high-sensitivity C-reactive protein) were assessed.

**Results:**

The range of IL-6 (pg/mL) in each tertile was T1 (0.71–4.16), T2 (4.20–7.84), and T3 (7.9–236.32). Compared with T1, patients in the highest IL-6 tertile were more commonly male (56% versus 35%) and had higher creatinine (117±45 versus 101±36 μmol/L), hsCRP (11.6 [4.9–26.6]mg/L versus 2.3[1.1–4.2] mg/L). In univariable analysis, rates of all-cause death, cardiovascular death, and sHFH were higher in T3 versus T1. All-cause and cardiovascular death rates remained higher in T3 versus T1 after adjustment (*P*<0.001). One log unit increase in IL-6 was associated with higher risk of all-cause death (hazard ratio, 1.46 [1.17–1.81]), cardiovascular death (hazard ratio, 1.40 [1.10–1.77]), and sHFH (hazard ratio, 1.24 [1.01–1.51]) after adjustment. One log unit increase in hsCRP was associated with a higher risk of cardiovascular death and all-cause death before and after adjustment for other factors but was not associated with risk of sHFH before or after adjustment.

**Conclusions:**

In patients recently hospitalized with heart failure with preserved ejection fraction, IL-6 is an independent predictor of all-cause mortality, cardiovascular death, and sHFH after adjustment for risk factors including BNP. These findings are of particular relevance in the context of current anti–IL-6 drug development.

**What is New?**

- In patients recently hospitalized with acute decompensated heart failure with preserved ejection fraction, IL (interleukin)-6 is predictive of all-cause mortality, cardiovascular death, and heart failure hospitalization after adjustment for clinical risk factors including BNP (B-type natriuretic peptide).
- Patients recently hospitalized with acute decompensated heart failure with preserved ejection fraction have high circulating levels of IL-6.
- The association between IL-6 and clinical outcomes appears to be stronger than these associations with CRP (C-reactive protein), the downstream product of IL-6.

**What Are the Clinical Implications?**

- These results highlight inflammation, and IL-6 in particular, as a potential therapeutic target in heart failure with preserved ejection fraction.
- Further consideration should be made to the development and use of IL-6 as a prognostic marker in clinical practice.

Inflammation may play a central role in the pathophysiology of heart failure with preserved ejection fraction (HFpEF).^1^ IL (interleukin)-6 is the principal stimulus to the hepatic synthesis of CRP (C-reactive protein) and high circulating concentrations of both IL-6 and CRP have been associated with the development of HFpEF.^2–5^ However, robust data specifically addressing the association between circulating levels of IL-6 and prognosis for patients with HFpEF are limited.^6–8^

The importance of the IL-6 signaling pathway in cardiovascular disease is increasingly recognized. More recently, it has become a major focus as a potential pathogenetic mediator and therapeutic target in patients with heart failure. Its role is best understood in relation to atherogenesis and it is implicated in the development of ventricular hypertrophy.^9–12^ Both of these processes are important in the cause of HFpEF. Higher circulating concentrations of IL-6 have been associated with the subsequent development of HFpEF.^3^ However, the majority of data pertaining to IL-6 (and IL-1/1β, its upstream signaling precursor) in prevalent heart failure are from patients with heart failure with reduced ejection fraction (HFrEF) or all-comers with heart failure in whom ejection fraction is undefined.^8,13–20^ The CANTOS trial (Canakinumab Anti-Inflammatory Thrombosis Outcome Study) reported that, in patients with a history of prior myocardial infarction and elevated hsCRP (high-sensitivity CRP), treatment with the IL-1β inhibitor, canakinumab, reduced heart failure hospitalization and heart failure–related mortality, although an examination of these effects specifically upon HFpEF was not addressed.^10,11^ ZEUS (Ziltivekimab Cardiovascular Outcomes Study) is a large phase 3 trial examining the effect of IL-6 inhibition upon cardiovascular outcomes in patients with chronic kidney disease and elevated hsCRP.^21^ This trial incorporates baseline and serial assessment of LVEF and includes heart failure events as an outcome measure.^21^

Given the noteworthy associations of HFpEF with coronary microvascular and macrovascular disease, ventricular hypertrophy, and inflammation, anti–IL-6 treatment may have particular relevance for patients with HFpEF. Furthermore, inflammatory activation may be elevated in the weeks following discharge after hospitalization for HFpEF. This period is well-recognized as a period of heightened risk for adverse outcomes, including rehospitalization and death.^22^

We examined the relationship between IL-6 and clinical outcomes in patients recently hospitalized with HFpEF.

## Methods

We enrolled near-consecutive patients admitted with a primary diagnosis of decompensated HF (irrespective of left ventricular ejection fraction [LVEF]) at 3 hospitals in the West of Scotland.^23^ HF was defined according to the criteria outlined by the European Society of Cardiology.^24^ The diagnosis of HF required typical symptoms and signs of heart failure. For the diagnosis of HFpEF, this required LVEF to be >40% with relevant structural heart disease (LV hypertrophy/left atrial enlargement) or diastolic dysfunction. Eligible patients were required to be 18 years of age or older and to have an elevated BNP (B-type natriuretic peptide >100 pg/mL). The main exclusion criteria were primary presentation with myocardial infarction or concurrent systemic disease likely to result in reduced life expectancy. Attendance for the study visit and measurement of inflammatory biomarkers took place 1 month after discharge from hospital. For the current analysis, we included only patients with LVEF >40%. This threshold was selected as it reflects the LVEF inclusion criterion used in recent major clinical trials in HFpEF and is consistent with cutoffs used in prior analyses of inflammatory markers in HFpEF.^8,25,26^ Of 1003 patients originally enrolled, 628 patients (65%) returned for the study visit. Failure to attend was due to death (n=115), deterioration in health (n=73), or withdrawal of consent (n=167). Three hundred seventeen patients had LVEF >40%. IL-6 data were unavailable for 31 patients. These 286 patients with HFpEF and IL-6 data comprise the current study population.

The study complied with the Declaration of Helsinki and was approved by the Local Ethics Committee. All patients provided written informed consent. All data provided are anonymized to respect the privacy of patients who have participated in line with applicable laws and regulations. The data will be provided upon reasonable request to the corresponding author.

### Left Ventricular Ejection Fraction

LVEF was measured by 2-dimensional echocardiography. Analysis was performed offline, using the biplane method of discs (modified Simpson rule) by a single operator blinded to patient information. As a result of inadequate endocardial border definition, it was not possible to measure LVEF in 26 patients and these patients were excluded from analysis.

### Laboratory Measurements

Whole blood was drawn by venepuncture. Samples were processed immediately by centrifugation at 3000*g* for 15 minutes and serum and plasma fractions were aliquoted for storage at −80 °C until assay. Immunoassays were used to measure IL-6, KIM-1 (kidney injury marker 1), TNF-α (tumor necrosis factor alpha; Singulex, Alameda, CA), and hsCRP (high-sensitivity C-reactive protein; Siemens BN II Nephelometer, Siemens Healthcare Diagnostics GmbH, Marburg, Germany). Plasma BNP was measured using the Abbott Architect assay (Abbott Diagnostics, Maidenhead, United Kingdom). hsTnI (high-sensitivity troponin I) was measured using the Architect assay (Abbott Laboratories, Abbott Park, IL). Galectin-3 was measured by ELISA (BG Medicine, Waltham, MA). All other biochemical and hematological assays were performed in local National Health Service laboratories in Glasgow, United Kingdom, and these assays all performed adequately in the relevant national external quality assurance schemes.

### Follow-Up

Outcomes were captured using routinely collected data. Patients were “flagged” using the Information Services Division of the Scottish Health Service data on hospital admissions as well as in-hospital and out-of-hospital deaths, held by the General Register Office for Scotland. Subsequent heart failure hospitalization was defined as any hospitalization with any of the following International Classification of Diseases codes: I110, I255, I420, I426, I427, I428, I429, I50, I500, I501, I509.

### Statistical Analysis

Patients were divided into tertiles according to IL-6 levels. Baseline characteristics are presented as frequencies and percentages for categorical variables and mean with SDs or medians with interquartile range for continuous variables. A nonparametric test for trend across groups, an extension of the Wilcoxon rank sum test, was used to examine for variation in baseline characteristics across IL-6 tertiles. All continuous variables were log-transformed as appropriate to normalize their distribution. The primary outcome (all-cause mortality), cardiovascular death, and subsequent heart failure hospitalization were analyzed for each tertile using Cox-regressions. Times to events are displayed using Kaplan-Meier curves according to tertile. Models were adjusted for validated clinical risk factors which included age, sex, creatinine, systolic BP, ejection fraction, BMI, diabetes, previous myocardial infarction, stroke, heart failure hospitalization before the enrollment episode, and also BNP at the time of enrollment. IL-6 and other additional relevant biomarkers were examined as continuous variables for all outcomes in both univariable and multivariable Cox regression models. Spearman correlation coefficients were examined where appropriate to evaluate the correlation between IL-6 and other clinically important variables. A restricted cubic spline of IL-6 was generated and displayed graphically using the xblc command in STATA. *P*<0.05 was considered statistically significant. All statistical analysis was performed using STATA version 16.0.

## Results

### Baseline Characteristics

Data from 286 participants with HFpEF were analyzed. Tertile ranges of IL-6 were as follows: tertile 1 (T1): 0.7 to 4.2 pg/mL, tertile 2 (T2): 4.2 to 7.8 pg/mL, and tertile 3 (T3): 7.9 to 236.3 pg/mL. Sixty-one percent of patients had concentrations of IL-6 that were greater than the previously reported 95th centile of normal values (4.45 pg/mL).^27^ Table 1 summarizes the baseline characteristics (at time of admission to hospital) of the patients in each tertile. Patients in tertile 3 (highest IL-6), compared with tertile 1 (lowest IL-6) were less likely to be female (45% versus 65%, *P*=0.005) and had higher serum creatinine (117±45 μmol/L versus 101±36 μmol/L, *P*=0.002). Cardiovascular treatment and the prevalence of the commonly encountered cardiac and noncardiac comorbidities including stroke, myocardial infarction, atrial fibrillation/flutter, diabetes, and chronic obstructive pulmonary disease were similar across the tertiles.

**Table 1.**
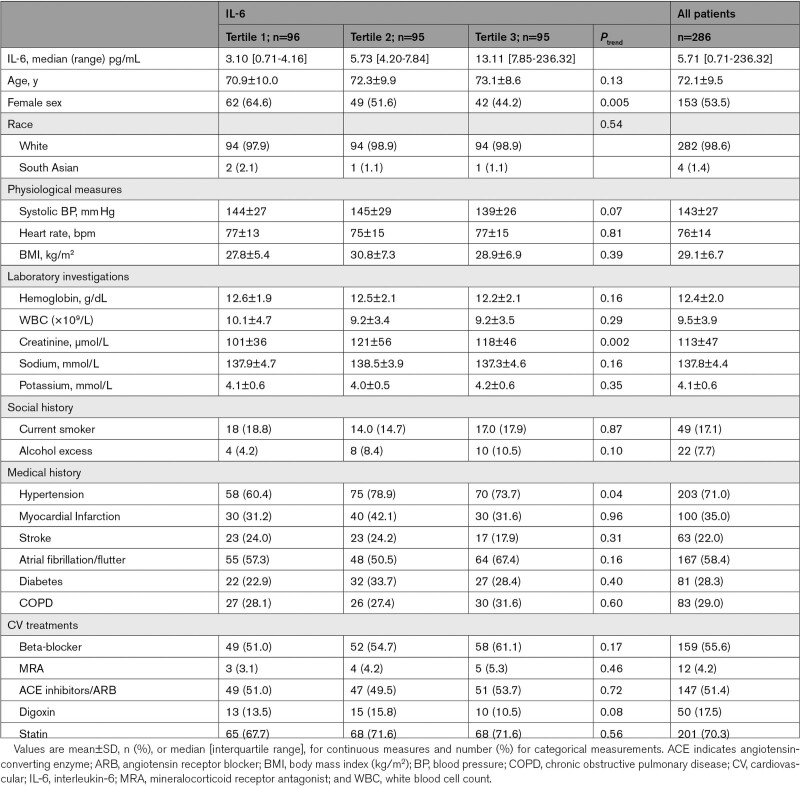
Baseline Characteristics According to IL-6 Tertiles

### Heart Failure Characteristics and Biomarkers

LVEF did not vary across IL-6 tertiles (Table 2). Although there was a trend towards higher BNP levels, this did not reach statistical significance (*P*=0.07), and nor did hsTnI concentrations vary significantly across tertiles of IL-6 (*P*=0.28). Markers of inflammation (TNF-α, hsCRP) and fibrosis (galectin-3) were higher in tertile 3 when compared with tertile 1 (*P*<0.05). Patients in tertile 3 had more peripheral edema when compared to patients in tertile 1.

**Table 2.**
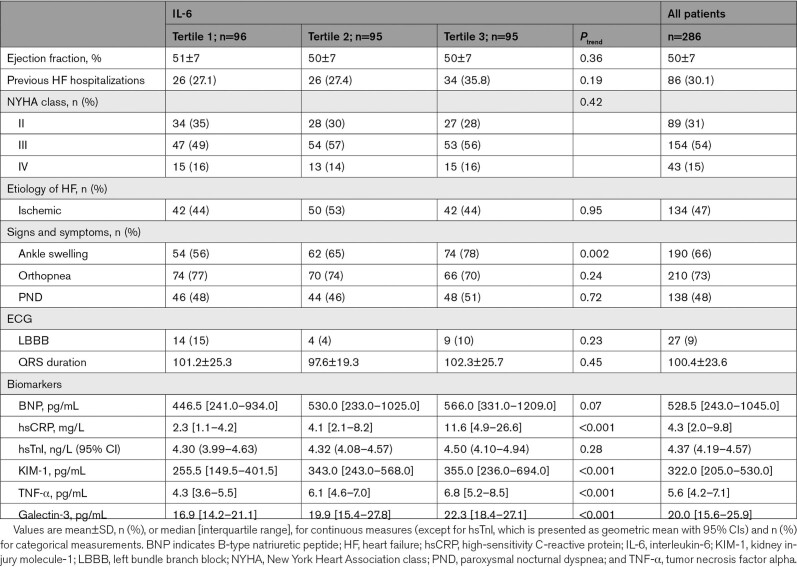
Heart Failure Characteristic According to IL-6 Tertiles

Levels of IL-6 correlated positively with circulating levels of hsCRP (r=0.57), TNF-α (r=0.41), and galectin-3 (r=0.33) but were only weakly correlated with creatinine (r=0.18) and KIM-1 (r=0.21). IL-6 did not correlate with BNP (r=0.09; Table 3).

**Table 3.**
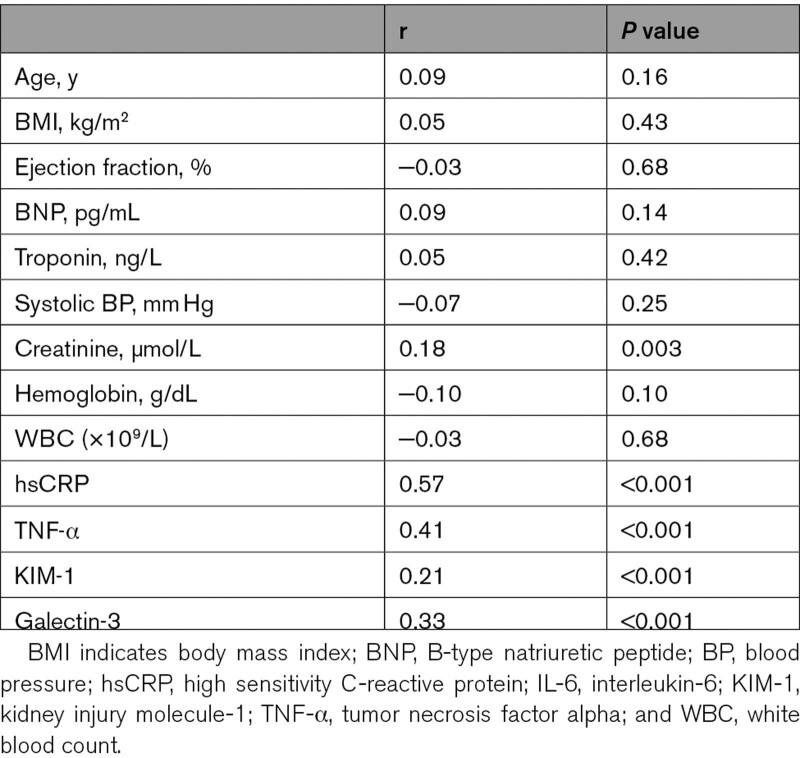
Spearman Correlation Between IL-6 and Clinical Variables and Biomarkers

### Association Between IL-6 and Clinical Outcomes

**Figure 1.**
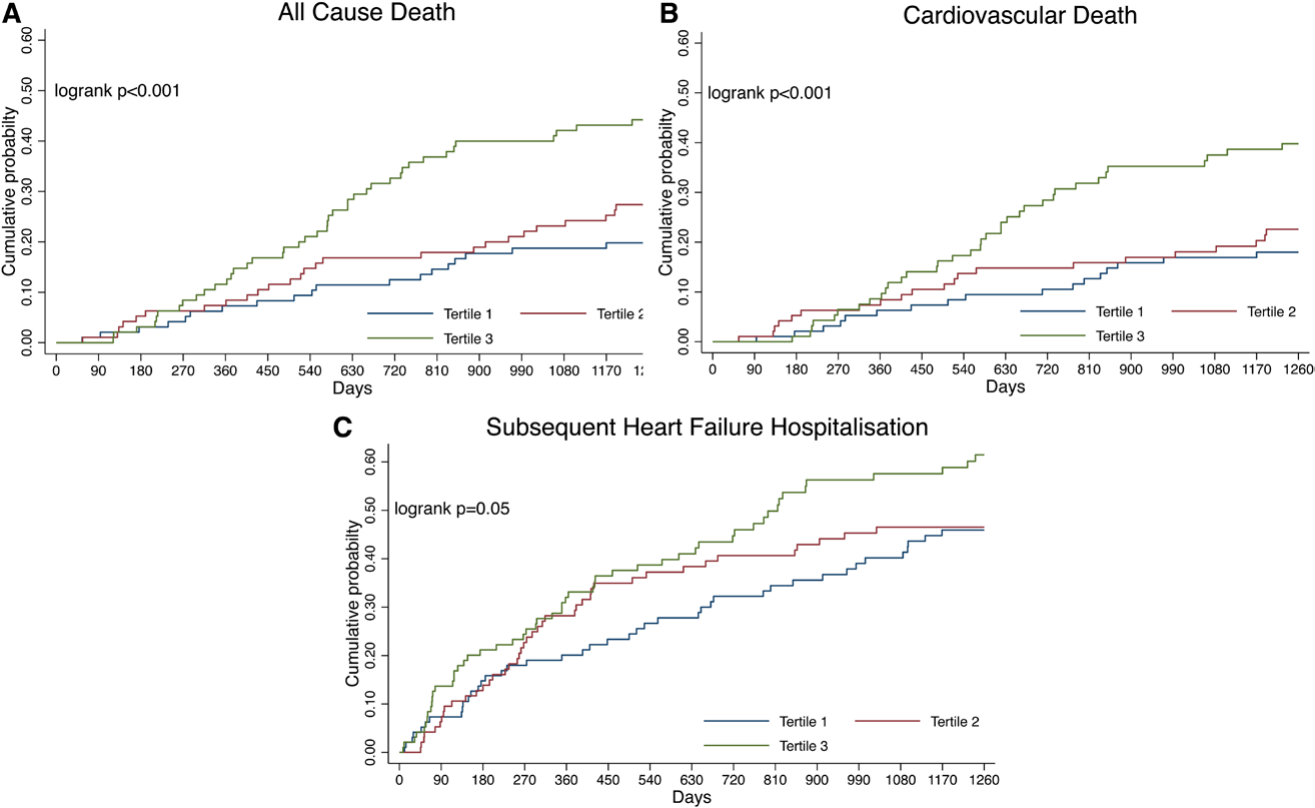
**Clinical outcomes for patients with heart failure with preserved ejection fraction according to IL (interleukin)-6 tertile. A**, Cumulative probability for all-cause death. **B**, Cumulative probability for cardiovascular death. **C**, Cumulative probability for subsequent heart failure hospitalization.

**Figure 2.**
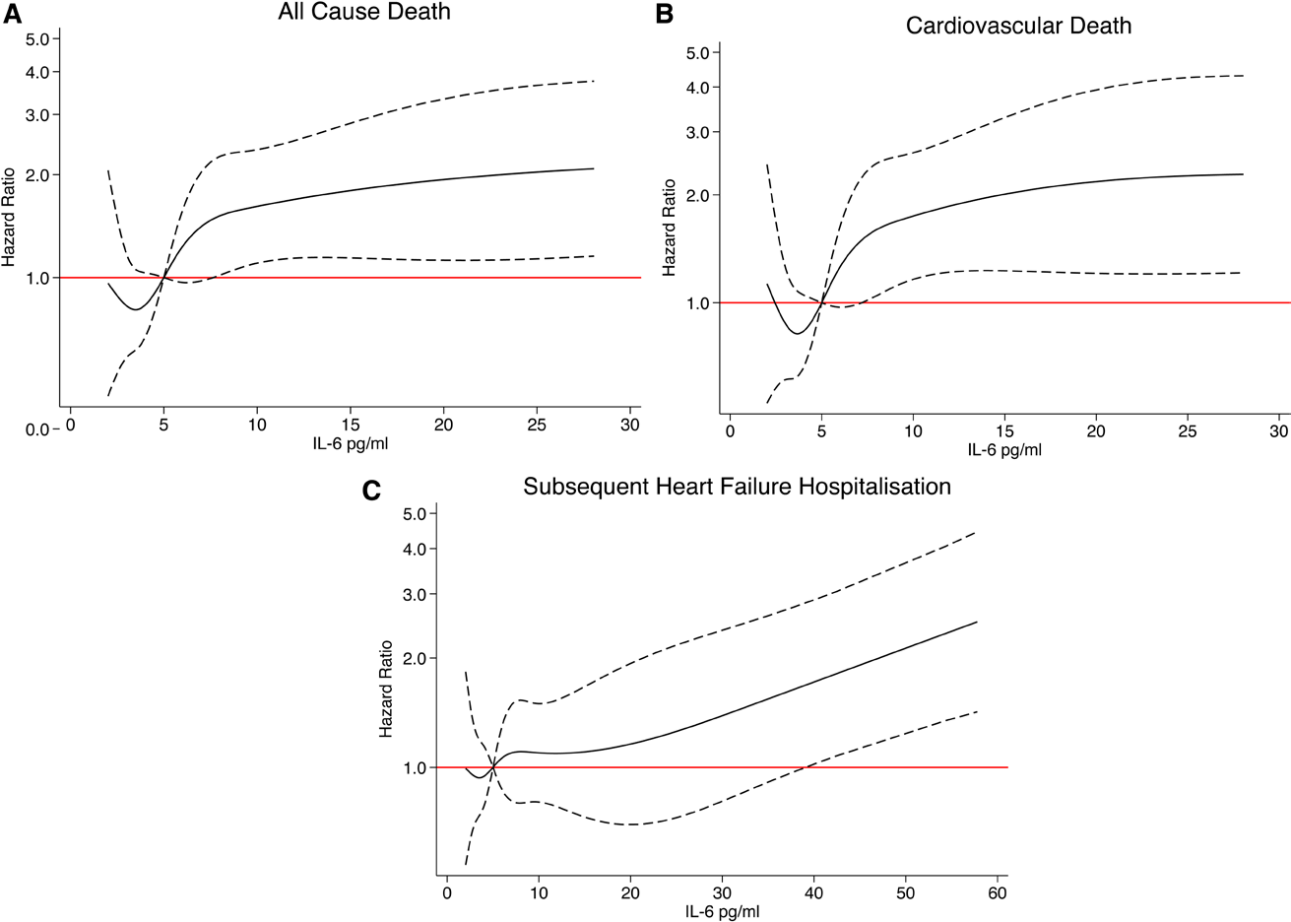
**Association between IL (interleukin)-6 levels and the risk of all-cause death, cardiovascular death, and subsequent heart failure hospitalization (restricted cubic spline analysis). A**, All-cause death. **B**, Cardiovascular death. **C**, Subsequent heart failure hospitalization. Model adjusted for age, female sex, systolic blood pressure, creatinine, body mass index, New York Heart Association (NYHA) class, diabetes, myocardial infarction, stroke, prior heart failure hospitalization, and log(B-type natriuretic peptide [BNP]).

The mean follow-up was 3.2±1.5 years, during which time 110 patients (39%) died. All-cause mortality occurred in 51 patients (18.2 per 100 patient-years) in tertile 3 and 27 patients (7.3 per 100 patient-years) in tertile 1 (Table 4 and Figure 1). The adjusted risk of all-cause mortality and cardiovascular death was higher in IL-6 tertile 3 when compared with tertile 1 (adjusted hazard ratio [HR] for all-cause mortality, 2.47 [95% CI, 1.49–4.11]; *P*<0.001) and cardiovascular death 2.46 (95% CI, 1.43–4.22, *P*<0.001). Although the risk of subsequent HF hospitalization was numerically higher for tertile 3 versus tertile 1, there was no increased risk of heart failure hospitalizations after adjustment. When assessed as a continuous variable and after adjustment, 1 log unit increase in IL-6 was associated with a higher risk of all-cause mortality (HR, 1.46 [1.17–1.81], *P*=0.001), cardiovascular death (HR, 1.40 [1.10–1.77]; *P*=0.005), and subsequent heart failure hospitalization (HR, 1.24 [1.01–1.51]; *P*=0.044; Table 5 and Figure 2).

**Table 4.**
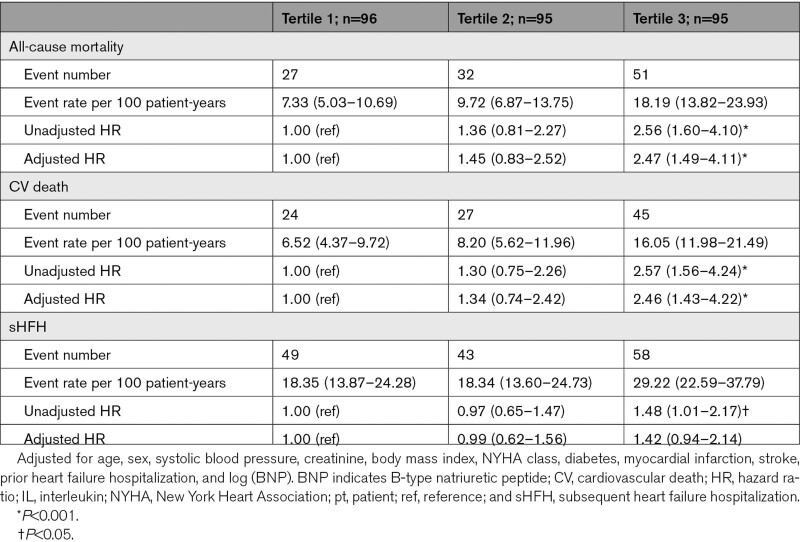
Outcomes According to IL-6 Tertiles

**Table 5.**
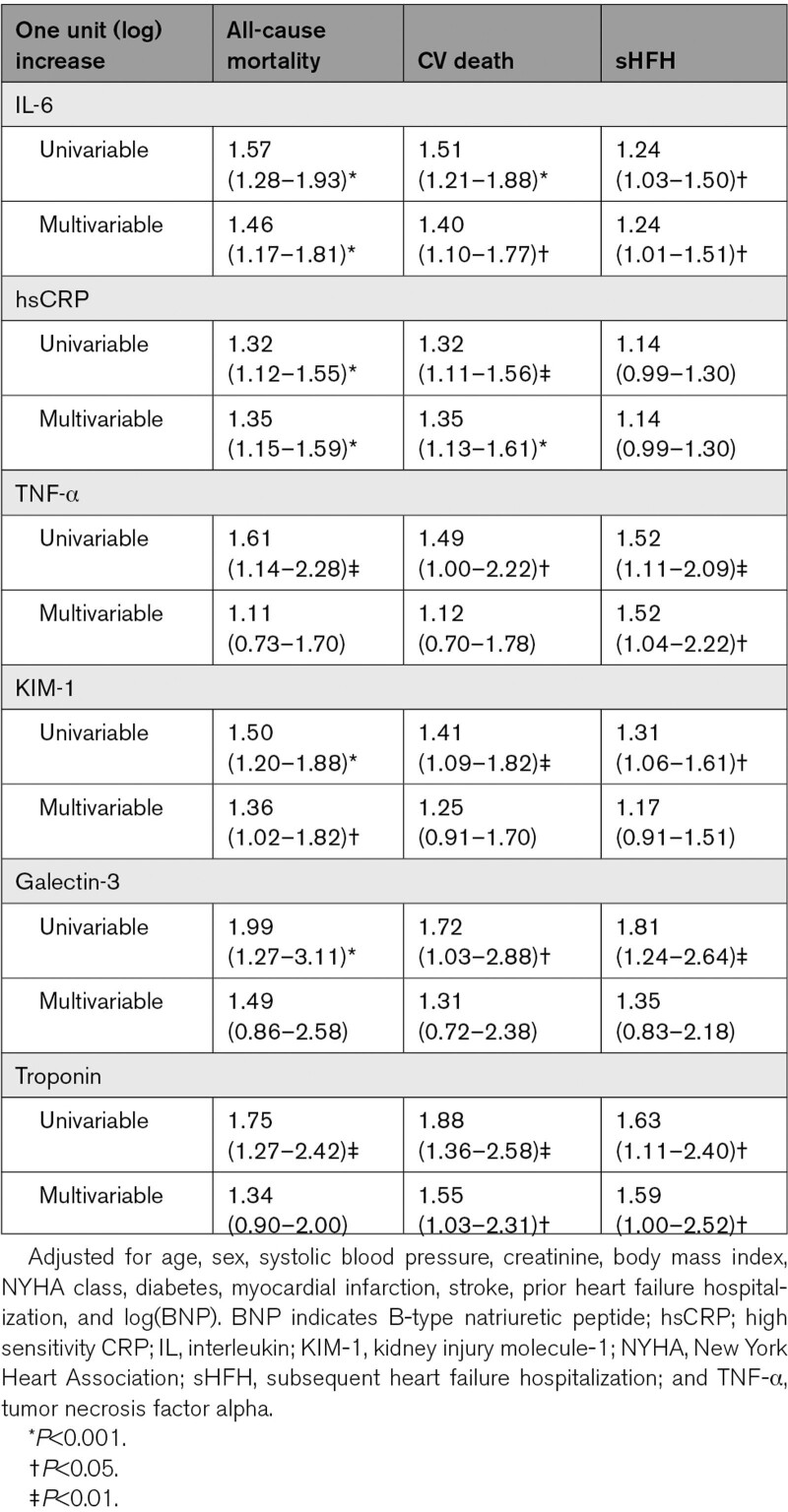
Univariable and Multivariable Analysis According to Other Biomarkers

### Association Between Other Biomarkers and Clinical Outcomes

When modeled as a continuous variable, 1 log unit increase of TNF-α, KIM-1, troponin, and galectin-3 was associated with higher risk of all-cause mortality, cardiovascular death, or subsequent heart failure hospitalization in univariable analysis (Table 5). After adjustment, hsCRP and KIM-1 remained associated with a higher risk of all-cause mortality. hsCRP was associated with a higher risk of cardiovascular death after adjustment but this association was not seen for TNF-α, KIM-1, or galectin-3 after adjustment. Troponin levels were also associated with a higher risk of cardiovascular death and subsequent heart failure hospitalization after adjustment but were not associated with all-cause mortality. Unlike IL-6, 1 log unit increase of hsCRP was not associated with subsequent heart failure hospitalization after adjustment. TNF-α was the only other biomarker associated with risk for subsequent heart failure hospitalization.

## Discussion

This is the first study to demonstrate the prognostic significance of circulating levels of IL-6 following hospital admission because of HFpEF. Patients with higher circulating concentrations of IL-6 were at an increased risk of all-cause death, cardiovascular death, and subsequent heart failure hospitalization. Importantly, we demonstrated that IL-6 remained an independent predictor of these events even after adjustment for established independent clinical risk factors including BNP. Therefore, our findings highlight that levels of IL-6 are not only important for the of development of HFpEF^3^ but also play a pivotal role in clinical outcomes.

By virtue of the enrolment of near-consecutive patients admitted to hospital with heart failure, the patients included in this analysis represent a typical cross-section of people admitted to hospital with HFpEF. Indeed, in keeping with a common HFpEF patient profile, the majority were elderly, over half were female and the prevalence of both hypertension and atrial fibrillation was high. In this high-risk population, 61% had a concentration of IL-6 that was greater than the previously reported 95th centile of the normal range (4.45 pg/mL) and hsCRP was ≥2 mg/dL in 77%, reflecting substantial inflammatory activity persisting 1-month post-hospital discharge.^10,27^ Even after adjustment for clinically important prognostic variables, including BNP, patients in the highest tertile of IL-6 concentration had over 2-fold increased risk for all-cause mortality and were at 2.8× higher risk for cardiovascular death during follow-up. Although the association between IL-6 tertile and subsequent heart failure hospitalization was not apparent after adjustment, when these outcomes were assessed in relation to IL-6 as a continuous variable, the association between higher IL-6 and heart failure hospitalization remained, even after adjustment. Indeed, each 1-unit log increase in IL-6 was associated with a 24% increased risk of subsequent heart failure hospitalization. IL-6 is therefore a marker of adverse outcomes in patients recently hospitalized with HFpEF.

IL-6 is an important pleiotropic cytokine that regulates the release of CRP and other acute-phase proteins. Therefore, it is not surprising that concentrations of IL-6 were correlated with its downstream product, hsCRP. However, in contrast to IL-6, hsCRP was not associated with risk for heart failure hospitalization. Furthermore, point estimates suggest that the increase in risk for all-cause and cardiovascular mortality was relatively greater for each log unit increase in IL-6 than it was for an equivalent log unit increase in hsCRP. IL-6 may, therefore, be of particular relevance in the stratification of patients for potential enrolment in trials of anti-inflammatory therapies, of particular relevance in the context of anti–IL-6 drug development. Although circulating concentrations of IL-6 also correlated with TNF-α, after multivariable adjustment, TNF-α was associated with subsequent heart failure hospitalization but not associated with all-cause or cardiovascular mortality. This finding may be of note in the context of previous neutral results from trials investigating anti-TNF-α drugs, including infliximab and etanercept, in the treatment of heart failure.^28,29^

Patients with the highest IL-6 concentrations had more frequent evidence of peripheral edema. Those patients may also have intestinal edema and increased permeability for gut endotoxins to enter the systemic circulation and evoke proinflammatory effects.^30^ Levels of IL-6 were higher in those with higher serum creatinine, which is in keeping with prior observations that higher levels of IL-6 are associated with deteriorating renal function. IL-6 may also play a role in sodium regulation.^31,32^ Increased levels of IL-6 were not associated with an increased prevalence of the common cardiac and noncardiac comorbidities such as diabetes and stroke. Notably, higher levels of IL-6 were also not associated with prior myocardial infarction. Although IL-6 has been clearly associated with atherogenesis, in patients with HFpEF, IL-6 may exert relatively more important pathophysiological effects in the promotion of endothelial dysfunction, coronary microvascular disease, and increased arterial stiffness.^33,34^ These processes are all relevant to the development and progression of HFpEF and the inflammatory hypothesis underlying the initiation and progression of HFpEF is increasingly well established.^1^ Although higher IL-6 levels were also associated with galectin-3, a biomarker related to the fibrotic effects of inflammation, in keeping with prior reports this marker was not associated with clinical outcomes after adjustment.^35–37^ In our cohort, KIM-1 was associated with higher risk of all-cause death after adjustment. KIM-1 is released from the proximal tubule of the kidney in response to metalloproteinase activity and has previously been associated with rehospitalization for heart failure after adjustment in an acute HFrEF cohort. However, it has not previously been explored in relation to outcomes for patients with HFpEF.^38–41^

The clinical significance of IL-6 in heart failure (HFrEF and HFpEF combined) was previously examined in the BIOSTAT-CHF (Biology Study to Tailored Treatment in Chronic Heart Failure) cohort.^8^ That multicenter observational study included patients from in-patient and out-patient settings, but only 10% of the cohort had HFpEF.^8^ Although poorer clinical outcomes were found in patients with IL-6 above the 95th centile, outcomes were reported for the cohort as a whole and associations were not specifically examined in patients with HFpEF in isolation.^8^ However, patients with HFpEF were 1.6× more likely to have IL-6 concentrations above 95th centile than patients with HFrEF. In samples obtained from 379 participants with HFpEF in the TOPCAT trial (Treatment of Preserved Cardiac Function Heart Failure with an Aldosterone Antagonist), a machine learning approach was used to examine multi-biomarker clusters to predict outcomes for patients with HFpEF.^7^ In that analysis, IL-6 was predictive of a composite of death and heart failure–related hospital admission.^7^ Unlike our study of recently hospitalized patients, those in TOPCAT were recruited from both in-patient and out-patient settings and were less symptomatic than patients in our study (38% of patients had class III/IV symptoms at enrolment in comparison to 69% of our patients). The number of events in this TOPCAT cohort was accordingly low, with only 94 events observed in 379 subjects over 2.9 years, in comparison to the 260 events (all-cause death and HF hospitalization) observed in our cohort.^7^ The biomarker assessment in the TOPCAT recruits used a multiplex analysis of 49 biomarkers, and the authors acknowledged that while providing informative data, this method has assay-specific limits of detection that may not be equivalent to those of established quantitative assays.^7^

We found that higher IL-6 levels were associated with adverse outcomes even after adjustment for BNP. Furthermore, IL-6 and BNP were not significantly correlated. These findings suggest the potential for additive, independent beneficial effects of anti-inflammatory therapies in the treatment of heart failure. This strategy would be complementary to current therapies with an emerging evidence basis for the treatment of HFpEF, including sodium-glucose cotransporter 2 inhibitors and also potentially angiotensin-neprilysin inhibition, whose primary therapeutic effects are not anti-inflammatory. Given the promising signal toward reduction in heart failure events seen in CANTOS and, particularly now with the large-scale clinical assessment of IL-6 inhibitors upon cardiovascular events, including heart failure across the spectrum of ejection fractions, better understanding of the association between IL-6 and cardiovascular outcomes has never been more relevant.^10,11^ Although elevated hsCRP has been used for trial entry, there may be a growing argument to use IL-6 thresholds.^42^ Personalization of therapy on the basis of elevated IL-6 as evidence of residual inflammatory risk, particularly in the high-risk posthospitalization period, remains an intriguing concept for exploration.

### Limitations

As with any study of this type, there are limitations to our work. The analyses described were retrospective and had not been prespecified. Only a single baseline measurement of IL-6 was available and this was measured ≈1 month after a hospitalization for HFpEF. However, the trajectory of IL-6 and other biomarker concentrations between hospital discharge and longer-term follow-up may be a valuable area for future research. The postdischarge assessments we assessed may be less susceptible to acute fluctuations during hospitalization. To date, no study has evaluated change in IL-6 levels over time in patients with HFpEF. However, in patients with acute HFrEF, a rise in IL-6 assessed 30 days after enrolment was associated with an increased risk of death.^43^ By enrolling patients at the time of heart failure hospitalization, we included a population that was more symptomatic and at higher risk of adverse outcomes than the general HFpEF population. However, we believe that these higher-risk patients are worthy of focused attention. Furthermore, recruitment of hospitalized patients allows greater confidence in the veracity of the HFpEF diagnosis which can be challenging, and particularly so in ambulatory patients whose condition may be relatively more confounded by noncardiac comorbidities, such as obesity, chronic lung disease, and physical deconditioning. Almost all of those included in our study were White patients, and the potentially important effect of race on outcomes and biomarkers has not been addressed here. LVEF was measured by a trained sonographer but, given the measurement error inherent to echocardiography, it is possible that some individuals may have had an LVEF<40%. Given the limitations of statistical power in the context of our sample size, we were unable to make further analyses stratified by LVEF above or below 50%.

### Conclusions

IL-6 is an independent predictor of all-cause mortality, cardiovascular death, and subsequent heart failure hospitalization in patients recently hospitalized with decompensated HFpEF. These findings highlight the adverse association between inflammation and outcomes for patients with HFpEF and are particularly pertinent in the context of anti–IL-6 drug development.

## Article Information

### Sources of Funding

This work was supported by The Scottish Executive Chief Scientist Office grant CZH/4/439 for the original study in which the patients were recruited. L. Mooney, M.C. Petrie, and Drs McMurray, Jhund, and Lang are supported by a British Heart Foundation Centre of Research Excellence Award (RE/18/6/34217).

### Disclosures

Dr Welsh reports receiving grants from Roche Diagnostics, AstraZeneca, Boehringer Ingelheim, Merck, Vifor, and AstraZeneca outside the submitted work. Dr Jhund received personal fees from Novartis and Cytokinetics and grants from Boehringer Ingelheim outside the submitted work. M.C. Petrie reports receiving grants and personal fees from Novartis, Novo Nordisk, AstraZeneca, Eli Lilly, Napp Pharmaceuticals, Takeda Pharmaceutical, Alnylam, Bayer, Resverlogix, Cardiorentis, and Boehringer Ingelheim outside the submitted work. Dr McMurray reports payments to his employer, Glasgow University, for work on clinical trials, consulting, lecturing, and other activities: Alnylam, Amgen, AstraZeneca, Bayer, Boehringer Ingelheim, Bristol Myers Squibb, Cardurion, Cytokinetics, Dal-Cor, GSK, Ionis, KBP Biosciences, Novartis, Pfizer, and Theracos; and personal lecture fees from Abbott, Hikma, Sun Pharmaceuticals, and Servier. Dr Lang received speaker’s fees from Roche, Pfizer, and Novartis and research grant support from Roche Diagnostics outside the submitted work. The other authors report no conflicts.
